# An Empirical Study on the Improvement of College Students’ Employability Based on University Factors

**DOI:** 10.3389/fpsyg.2022.793492

**Published:** 2022-03-07

**Authors:** Yi-Cheng Zhang, Yang Zhang, Xue-li Xiong, Jia-Bao Liu, Rong-Bing Zhai

**Affiliations:** ^1^School of Business, Anhui Xinhua University, Hefei, China; ^2^School of Management, University of Science and Technology of China, Hefei, China; ^3^School of Electronic Commerce, Hefei Information Technology University, Hefei, China; ^4^Master of Public Administration, University of Science and Technology of China, Hefei, China; ^5^School of Mathematics and Physics, Anhui Jianzhu University, Hefei, China

**Keywords:** course setting, course teaching, club activities, employment guidance, college students’ employability

## Abstract

With the popularization of higher education and the promotion of college enrollment expansion, the number of college graduates increases sharply. At the same time, the continuous transformation and upgrading of the industrial structure put forward higher requirements on the employability of college students, which leads to the imbalance between supply and demand in the labor market. The key to dealing with employment difficulties lie in the improvement of college students’ employability. Therefore, we make a regression analysis of 263 valid samples from universities in Anhui Province and extract the factors that influence the improvement of college students’ employability in the process of talent cultivation in university. The result shows that there is a positive correlation between course setting, course teaching, club activities, and college students’ employability, among which the course teaching and club activities are the most critical factors which may influence college students’ employability. In addition, from the viewpoint of individual college students, the overall grades of college students and the time of participating in the internship are also closely related to their employability, i.e., college students with good overall grades and long internship time should also have stronger employability.

## Introduction

Along with the popularization of higher education and the promotion of college expansion, the number of college graduates in China has shown explosive growth. According to statistics, the number of college graduates in China will be as high as 8.74 million in 2020 (Source: China Statistical Yearbook 2020). At the same time, the transformation and upgrading of China’s industrial structure and the improvement of production methods have put forward newer and higher requirements for the quality and ability of college graduates. Therefore, an “employment gap” has been left in the labor market, i.e., college graduates cannot find suitable jobs and enterprises cannot recruit suitable talents. To solve the employment difficulties of college graduates, it is crucial to improve the employability of college students. Employability is an important element of talent training in universities and a key indicator of the quality of higher education training, and a core competency that the supply side of the labor market should have (Abd Majid et al., 2020; Misni et al., 2020). Qian Weichang, an academician of the Chinese Academy of Sciences, held the opinion that higher education institutions should focus on cultivating college students’ self-learning ability, practical ability, and the ability to acquire knowledge and that efficient teachers are crucial in the teaching process (Chen, 2020). Therefore, based on the perspective of the university, we explore the factors influencing the employability of college students. It is of great practical significance to enhance the employability of college students and improve the difficult employment situation.

## Literature Review

### Employability Study

Up to now, scholars have not reached an academic consensus on the definition of employability, and the representative view is that employability is a person’s ability to obtain a job and become employed, maintain employment, and re-enter employment for various reasons, including factors affecting employability such as knowledge, skills, and attitudes (Hillage and Pollard, 1998; Harvey, 2001). College students can improve their employability through independent study and social practice (Meyer and Benguerna, 2019; Pitan and Muller, 2019). With the rapid development of the global economy, talent is becoming an increasingly scarce resource, which has contributed to the general focus on employability in twenty-first century society (Nicholas, 2018; Pitan and Muller, 2019).

### Employability Structure

The denotation and connotation of “employability structure” have not been clearly defined by scholars. The more recognized USEM model, proposed by the British scholar’s Knight and Yorke, includes comprehension of professional and subject knowledge, acquisition of professional and general skills required for the job, and self-efficacy including personal qualities, self-confidence, enjoyment of learning, and metacognition that reflects strategic responses and thinking (Small et al., 2018). Zheng (2002), a professor at Tsinghua University, classified college students’ employability into five dimensions: learning ability, thinking ability, practical ability, job application ability, and adaptability. Based on the USEM model, Shi and Wang (2018) proposed that employability includes four aspects: subject comprehension, basic skills, self-efficacy, and metacognition. This study proposes the composition structure of college students’ employability, including professional knowledge and skills, learning ability, adaptability, practical ability, communication ability, teamwork ability, information acquisition ability, and career planning ability.

### Factors Influencing Employability

There are also many studies on the factors influencing the employability of college students. Some scholars believed that employability has a strong correlation with individual factors. For example, Zhu (2014) investigated 327 high-tech enterprises in Zhejiang Province as a sample and used the *t*-test to conclude that college students’ employability is closely related to individual learning ability and innovation ability. Peng (2014) investigated the graduates of five colleges and universities in Nanchang, empirically analyzed the influencing factors of college students’ employability by using econometric modeling, and found that social practice activities and work practice had a significant positive influence on college students’ employability. Lu et al. (2017) investigated college students in six universities in Tianjin and used the structural equation modeling method to reveal the factors influencing college students’ employability, and confirmed that personal traits, professional quality, and social adaptability had a strong positive relationship with college students’ employability, while basic skills had a positive relationship with college students’ professional quality. Other scholars argued that employability is closely related to the factors of colleges. For example, Tan (2014) combed through the relevant literature and pointed out that the key point to enhance the employability of college students is the higher education model. Based on the CareerEDGE model, Tang et al. (2015) surveyed 236 fresh university graduates in economics and management, 101 staff members of employers, and 89 university faculty members, and found that there are five core factors influencing graduates’ employability, such as “strong ability to adapt to the environment” and two short-term factors influencing job search, such as “self-presentation ability.” The survey shows that there are differences in the perceptions of students’ employability among university teachers, students, and employers. Zhang et al. (2018) empirically analyzed the influence of the cultivation mechanism of colleges and universities on the employability of college students by establishing a mathematical model.

In summary, scholars have studied the concept of employability of college students and the influencing mechanism from different perspectives and achieved certain research outcomes. However, by combing through the literature, we find some limitations. First, employability dimensions generally lack a theoretical foundation, and the logical relationship between employability dimensions and definitions of employability is not strong. Second, the conceptualization of college students’ employability is inconsistent, and studies on information acquisition and career planning skills in the employability dimension are rarely seen. Third, the antecedent variables of college students’ employability are inconsistent, and few studies consider the factors of club activities and employment guidance. Fourth, although some scholars pointed out that higher education influences college students’ employability, most of them focused on theoretical elaboration, few of them used empirical analysis to verify whether there is a positive correlation between higher education and college students’ employability and what college factors have a more significant effect on improving college students’ employability. Therefore, based on the perspective of colleges and universities, this article proposes four factors that affect college students’ employability: course setting, course teaching, club activities, and employment guidance. By obtaining data through questionnaires, we empirically analyze the correlation between the aforesaid factors and college students’ employability and investigate which factor has the most critical influence on improving college students’ employability.

## Theoretical Background and Research Hypothesis

In this study, we divide the college factors into four aspects: course setting, course teaching, club activities, and employment guidance, and analyze their effects on the improvement of college students’ employability.

### Prediction of Course Setting on College Students’ Employability

Human capital theory suggests that the increase in human capital, such as human knowledge and skills, far exceeds the contribution of labor resources to social and economic development. Education is the most effective investment for individual capital appreciation, and by continuously acquiring knowledge in learning and practice, one can effectively improve his employability (Leonardi and Chertkovskaya, 2017). Therefore, the reasonable setting, of course, is an effective way to improve the input-output ratio of college students’ human capital, which has an important impact on the employability of college students. Considering the above background, we put forward the following hypotheses.

Hypothesis 1: The rationality of college course setting has a positive contribution to the improvement of college students’ employability.

### Prediction of Course Teaching on College Students’ Employability

The course setting is the teaching plan of universities, while course teaching is the implementation of the teaching plan. The key to course teaching lies in the enhancement of teaching quality and the investment of teachers’ time and energy. For one thing, the quality of teaching in college courses is fundamental to the improvement of students’ employability, and some studies have shown that the stricter the quality control of teaching, the more knowledge students acquire (Wu et al., 2019). For another, the more time and energy teachers invest in teaching (e.g., helping students answer questions and solve problems, and handling student assignments on time), the better they can promote the teacher-student interaction, which can stimulate students’ interest in learning and improve their independent learning. Garnett (2014) have shown that the more enthusiasm teachers invest in teaching and the more time they spend helping students answer questions and solve problems, the more knowledge and skills students will acquire. Based on the previous discussion, we put forward the following hypotheses.

Hypothesis 2: The teaching of college courses has a positive contribution to the improvement of college students’ employability.

### Prediction of Club Activities on College Students’ Employability

Club activities are an important form of practical education for college students and a complement to theoretical education in universities. Social capital theory suggests that people acquire tangible and intangible resources at the individual, group, and organizational levels through social interactions and connections with others. One of the key ideas of the theory is that social capital resources are embedded in the social networks of interconnected individuals, groups, or organizations and can be accessed through social networks of relationships. As a social organization on campus, the college club assumes the role of building bridges for communication and exchange. Therefore, college clubs can be seen as a kind of social capital. Club activities can provide more opportunities for students to communicate and exchange ideas, and bring them a stable social relationship. When college students participate in social activities, they can deepen their understanding of theoretical knowledge and transform it into practical skills for work. Furthermore, they can strengthen their communication with others and enhance their sense of cooperation. Some studies have shown that college students who regularly participate in club activities have better communication skills (Buckley and Lee, 2021). In addition, participation in club activities, especially college-enterprise cooperation, gives college students more opportunities to get in touch with the outside society and know more about job-hunting, corporate recruitment needs, and the workplace environment. By having extensive contact and communication with society and making full use of the information gained in the job-hunting process, college students can increase their success rate in employment. From the above discussion, we put forward the following hypothesis.

Hypothesis 3: College club activities have a positive contribution to the improvement of college students’ employability.

### Prediction of Employment Guidance on College Students’ Employability

Employment guidance includes a series of employment services, such as career training courses, career seminars, CV guidance, and so on, provided by colleges to enhance the success rate of employment. Nowadays, colleges have set up career planning offices and employment guidance centers. The influence of the establishment of employment guidance institutions on the employability of college students also belongs to the research scope of social capital theory. Social capital theory suggests that individuals exist in social networks and have distinctly strong and weak relationships with members of the organizations in the network. Strong relationships in networks refer to a strong homogeneity of network members, i.e., a convergence of opinions held by groups of people in social interactions, a close relationship between people, and a strong emotional element that sustains them. Therefore, strong relationships are a very favorable channel for job hunting. The counterpart to strong relationships is weak relationships, and although they are less stable than strong relationships, they are broader in scope and still have a more important impact on the job-hunting channel.

Currently, most college students obtain job-hunting information through the Internet or the job market. Although this formal channel can bring more job opportunities for college students, there are some specific problems such as low employment quality, rate, and efficiency. Conducting employment guidance activities is an important way of making recommendations for college students, aiming at providing high-quality employment information and broadening their employment channels. This informal job search channel can provide college students with high-quality employment opportunities (Pitan and Atiku, 2017; Lu, 2019; Okolie et al., 2020). We put forward the following hypotheses.

Hypothesis 4: College employment guidance has a positive impact on the improvement of college students’ employability

## Research Design and Results Analysis

### Questionnaire Design and Data Collection

To ensure the credibility of the questionnaire, the questionnaire design was analyzed in detail before the survey, and scholars were invited to revise the presentation and wording of the questionnaire. To ensure the reliability of the questionnaire, a small-scale preliminary test (exploratory factor analysis) was conducted, the questionnaire was analyzed and revised to address the issues revealed during the test.

After the pilot test, the authors started the formal questionnaire survey. To ensure the validity and timeliness of the questionnaire distribution, we used the resources of the college and distributed paper questionnaires in different colleges. Meanwhile, we also handed out the questionnaire online by using the “Wenjuanxing” platform. The time span of the questionnaire distribution was from August to November 2020, which lasted for 3 months in total. In this article, undergraduates from Anhui provincial universities, namely, Anhui University, Hefei University of Technology, and Hefei College, were the main research subjects (sample data). A total of 1,000 college students were selected as the sample of this study. A total of 293 questionnaires were collected, and 266 valid responses were obtained by eliminating those with missing values or irregularities, resulting in a valid response rate of 26.6%. To ensure that this study was not affected by non-response bias, we divided the responses into two parts, including early responses and late responses, and compared the differences in age, gender, and family background between the two parts of the questionnaire respondents. The results showed that there was no non-response bias.

### Variable Measurement and Reliability and Validity

Reliability refers to the degree of consistency in the results obtained when the same method is used to measure the same object repeatedly. Validity refers to the degree to which a measurement instrument can accurately measure something, and the higher the validity, the better the item reflects the content being examined. Therefore, to accurately analyze the influence of the four factors of course setting, course teaching, club activities, and employment guidance on students’ employability, we need to conduct reliability and validity analyses on the data obtained from the questionnaire. All variables in the questionnaire were measured by 5-point Likert scales ranging from 1 (strongly disagree) to 5 (strongly agree). The factor load of each variable was obtained by Anderson-Rubin’s factor analysis method, and the reliability threshold was used to represent the scale reliability (α > 0.6).

#### Employability

As there is no standardized scale for measuring employability, this article draws on the mainstream literature (McQuaid, 2005) and summarizes previous research to propose a scale for measuring employability. The scale consists of eight secondary dimensions: professional knowledge and skills, learning ability, strain ability, practical ability, communication ability, teamwork ability, information acquisition ability, and career planning ability, which can truly reflect both the employability and overall quality of college students. To further ensure that the employability scale can reflect the employability of college students accurately, 10 college students were invited to be interviewed, after the first batch of questionnaire revision, and analyzed and verified the validity of the employability scale.

The abovementioned 10 respondents assessed each of the employability questions on a scale of 1–4 (1 = not relevant, 2 = somewhat relevant, 3 = very relevant, and 4 = highly relevant). Subsequently, in this article, the content validity index (CVI) is used to assess the validity of the employability scale by judging the results given by the college students interviewed (Sirén et al., 2012).

In terms of validity, the CVI values for each of the eight secondary-level dimensions of the employability scale were above the set threshold of 0.8. In terms of reliability, the Cronbach’s alphas for all eight secondary-level dimensions of the employability scale were greater than 0.7, and the overall Cronbach’s alphas for the scale was 0.823. The factor loadings were in the range of 0.606–0.806 which exceeds the minimum threshold of 0.6 (Sirén et al., 2012). The previous data indicate that the reliability of the employability scale is satisfactory. The employability scale is shown in Table 1.

**TABLE 1:** Employability scale.

Variable name	Number	Item	Cronbach’s alphas
Professional knowledge and skills	1	Communicate with others in English fluently	0.731
	2	Knowledge of laws and regulations	
	3	My professional knowledge and skills are firmly mastered	
	4	I have obtained professional certificates of computer	
Learning ability	1	In the conflict between study and entertainment, I will give priority to study.	0.828
	2	Ability to apply professional knowledge and skills.	
	3	I can learn without supervision.	
	4	I can study hard even if I do not like the course.	
Strain ability	1	No matter how the external environment changes, I can adapt quickly.	0.799
	2	I can handle emergencies with ease.	
	3	I am able to adjust my mindset in time.	
Communication ability	1	I can always communicate well with others.	0.808
	2	I can organize my words and express my ideas accurately.	
	3	I can understand other people’s feelings and communicate emotionally.	
Practical ability	1	I can effectively use my knowledge, skills and experience to solve problems.	0.882
	2	I can flexibly use what I have learned to deal with problems.	
	3	I can learn from experience and lessons in practice.	
	4	When encountering difficulties, I can combine my professional knowledge to deal with them.	
Teamwork ability	1	I can solve tough problems as a team.	0.836
	2	In order to ensure the smooth solution of the problems, I will seek the opinions and suggestions of team members.	
	3	I will effectively cooperate with the team to complete the task.	
Information acquisition ability	1	Access to information through multiple channels.	0.897
	2	Be able to accurately grasp the key to information.	
	3	Ability to listen attentively and capture important information of a conversation.	
	4	Can filter out false information in the mass of information.	
	5	Ability to quickly and comprehensively analyze information.	
Career planning ability	1	It is believed that good career planning ability can improve the success rate of job-hunting.	0.802
	2	Have a clear plan for your future career development.	
	3	Have a career plan that promotes self-development.	

#### Course Setting

Based on the planning of the college course setting, referencing the literature (McLean et al., 2016), this article adopts ten question items to measure the overall situation of the course setting. The questions evaluate the rationality of the course setting in terms of its specialization, knowledge coverage, course system, and course frontiers. The Cronbach’s alpha of the scale was 0.946 and the range of factor loadings was 0.691–0.831. The data showed that the reliability of the course setting scale was satisfactory. The course setting scale is shown in Table 2.

**TABLE 2:** Course setting scale.

Variable name	Number	Item
Course setting	1	The scope of knowledge covered by specialized courses.
	2	The degree of implementation of practical lessons.
	3	The degree of crossover and integration between specialized disciplines.
	4	The reflection of English instruction to the demand of current social employability.
	5	The degree of emphasis on the combination of theory and practice in practice courses.
	6	The amount of network equipment and the openness of the computer lab.
	7	The degree of enrichment of professional courses and basic theories.
	8	The effectiveness of practical courses in improving comprehensive ability.
	9	The utilization of laboratories and training grounds.
	10	The content of specialized courses is related to the development of the discipline and the cutting-edge trend.

#### Course Teaching

Based on the current situation of college course teaching and the relevant literature (McLean et al., 2016), this article adopts six questions to measure the basic situation of course teaching. The relatively comprehensive question items evaluate the rationality of course teaching in terms of the instructor’s commitment to the course, efficiency in dealing with problems, and the instructor’s length of service. The Cronbach’s alphas of the scale were 0.908 and the factor loadings ranged from 0.676 to 0.856. The data results showed that the reliability of the course teaching scale was qualified, and the course teaching scale is shown in Table 3.

**TABLE 3:** Course teaching scale.

Variable name	Number	Item
Course teaching	1	Teachers’ stimulation of students’ interest in learning during the teaching process.
	2	The attitude and efficiency of college teachers in dealing with teaching accidents.
	3	Effective use of teaching cases in the course of teaching.
	4	The timeliness of teachers’ correcting of and feedback on students’ homework.
	5	The number of years of English and computer teaching.
	6	Teachers’ commitment to teaching.

#### Club Activities

Based on the performance of college students’ club activities and the reference literature (Graupensperger et al., 2020), this article adopts four questions to measure the basic situation of college club activities. The question items assess club activities in terms of the diversity and importance of clubs. The Cronbach’s alphas of the scale were 0.884 and the range of factor loadings was 0.812–0.898. The data results showed that the reliability of the club activity scale was satisfactory and the club activity scale is shown in Table 4.

**TABLE 4:** Club activities scale.

Variable name	Number	Item
Club activity	1	The effectiveness of the college’s club activities.
	2	The diversity of club organizations.
	3	The college’s support of and emphasis on club activities
	4	Effectiveness of club activities in improving the corresponding competencies.

#### Employment Guidance

Based on the performance of college employment guidance and concerning relevant literature (Andrews and Higson, 2008), this article adopts four question items to measure the basic situation of employment guidance. The question items assess employment guidance in terms of the assessment methods of employment guidance, the soundness of career service providers, and career services. The Cronbach’s alphas for the scale were 0.889 and the factor loadings ranged from 0.788 to 0.859. The results of the data indicated that the reliability of the employment guidance scale was satisfactory. The employment guidance scale is shown in Table 5.

**TABLE 5:** Employment guidance scale.

Variable name	Number	Item
Employment guidance	1	Assessment methods for employment guidance courses.
	2	Soundness and completeness of the employment service institutions.
	3	Publicity and interpretation of the college’s employment situation and employment policies.
	4	Guidance on interview techniques and CV preparation.

#### Control Variables

To control the possible endogeneity problems in the study and to enhance the robustness of the findings, individual college students are introduced as control variables in this article. Previous research has shown that college students as individuals have some specificities, such as whether they are class leaders or not, and their grade ranking, which can affect their performance in future employment. Therefore, in this article, the student’s family background, overall grade ranking, internship duration, and class cadre status are used as control variables. The control variables of class cadre status are coded through dummy variables, where 1 means being a class cadre and 0 means not being a class cadre (class cadre status is used as the baseline variable for not being a class cadre). Overall grade ranking, family status, and internship status are measured using continuous variables.

### Data Analysis

#### Descriptive Statistics

Table 6 shows the descriptive statistics of the survey sample information. From Table 6, it can be seen that 92.1% of the sample members are under 25 years old. A total of 41.4% of the respondents were male and 58.6% of the respondents were female. Of the 266 graduates surveyed, 47% had an overall ranking in the top 25% of their class, 36% had an overall ranking between 25 and 50% of their class, 16.5% had an overall ranking between 50 and 75% of their class, and only 4.9% had an overall ranking below 75%. In addition, 56.8% of the students were class cadre at colleges.

**TABLE 6:** Sample description.

	Characteristic	Frequency	%
Score ranking	The top 25%	125	47
	25–50%	84	31.6
	50–75%	44	16.5
	Less than 75%	13	4.9
Gender	Male	110	41.4
	Female	156	58.6
Family background	Poor	44	16.5
	General	207	77.8
	Good	15	5.6
Class cadre	Yes	151	56.8
	No	115	43.2
Major	Arts	159	59.8
	Science	107	40.2

#### Validity and Correlation

In this article, we further analyzed the validity of each variable based on the reliability of the questionnaire. Validity is divided into convergent validity and discriminative validity, the former referring to the degree of similarity of the same feature measured by different measurement methods, and the latter indicating that when different methods are used to measure different constructs, the observed values should be able to be distinguished. First, to test the convergent validity of all the independent and dependent variables, this article used the statistical software PLS to test the average variance extracted (AVE) values of each variable. The results of the test showed that the AVE values of all variables were above 0.6, which was higher than the minimum threshold of AVE, indicating that the aggregated validity of each variable was good. Second, the discriminant validity of the variables was tested in two ways: the competitive model and the pairing combination method. The competitive model combines four independent variables and one dependent variable randomly into single-factor, two-factor, three-factor, four-factor, and five-factor models, and compares the goodness of fit of different factor models. The results in Table 7 show that the goodness of fit indicators of the five-factor model are better than those of other factor models, which indicates that the goodness of fit of the model separated from each variable is the best. From the perspective of the pairing combination method, this study conducted arbitrary pairing for five major variables and used the chi-squared test to distinguish the goodness of fit between different pairing models. Two models are established for each pair of variables by the structural equation model: the restrictive model with fixed covariance of 1 and the non-restrictive model without fixed covariance. The results showed that the fitting index of the unconstrained model was significantly better than that of the constrained model (*P* < 0.001). At the same time, PLS fitting test results of the structural equation model showed that the fitting degree of the model was good (*x*^2^/*df* = 1.821, *CFI* = 0.943, *TLI* = 0.924, *SRMR* = 0.042, *RMSEA* = 0.045). To sum up, the variables involved in this article have good validity.

**TABLE 7:** Discriminant validity of constructs.

Model	χ^2^/*df*	RMSEA	CFI	TLI	SRMR
Five-factor	1.821	0.045	0.943	0.924	0.042
Four-factors	2.321	0.077	0.816	0.842	0.061
Three-factors	2.509	0.083	0.775	0.767	0.064
Two-factors	2.769	0.085	0.731	0.718	0.067
Single-factors	2.456	0.089	0.711	0.702	0.070

Based on the above research, this article analyzed the correlation between variables. Table 8 shows that there is a significant correlation between course setting, course teaching, club activities, employment guidance, and employability. This result tentatively verifies that course setting, course teaching, club activities, and employment guidance have a positive impact on employability. In addition, the results show that there is a significant correlation between variables, and the correlation coefficients are all lower than 0.6, which indicates that there is no multicollinearity problem in the analysis process of hypothesis testing in this study.

**TABLE 8:** Correlation between variables (*N* = 266).

	Average	Variance	1	2	3	4
1 Course setting	3.847	0.6902				
2 Course teaching	3.868	0.7235	0.325**			
3 Club activity	3.769	0.7453	0.439**	0.451**		
4 Employment guidance	3.844	0.7770	0.476**	0.378**	0.366**	
5 Employability	3.737	0.5779	0.446**	0.434**	0.342*	0.396***

*p < 0.05, **p < 0.01, ***p < 0.001.

#### Hypothesis Testing

To objectively and accurately analyze the influence of college factors on the employability of college students, we added four control variables: family situation, overall grades, internship duration, and class cadre. The impact of different college factors on the employability of college students was analyzed. The specific results of the regression analysis are given in Table 9.

**TABLE 9:** Regression analysis.

Variable	Model 1	Model 2	Model 3	Model 4	Model 5	Model 6
Internship duration	0.281**	0.101*	0.092*	0.101*	0.089*	0.081
Class cadre	0.124	0.098	0.088	0.079	0.081	0.077
Overall grades	0.113*	0.084*	0.099*	0.102*	0.096*	0.101*
Family background	0.063	0.059	0.047	0.058	0.066	0.045
Master variable						
Course setting		0.272**				0.252**
Course teaching			0.311**			0.301**
Club activity				0.304**		0.289**
Employment guidance					0.289**	0.264**
*R* ^2^	0.142	0.199	0.221	0.211	0.204	0.206
Adjustment of *R*^2^	0.092	0.176	0.193	0.181	0.174	0.188
*F*	4.8732**	24.5672**	28.6793*	26.8432**	19.3827**	27.567**

The dependent variable for all the models in the table is the employability of college students. *p < 0.05, **p < 0.01.

First, the control variables were added to Model 1, and the results showed that internship duration (β = 0.281, *P* < 0.01) and overall grades (β = 0.113, *P* < 0.01) had a positive effect on the employability of college students, and the effective coefficient of internship duration was greater than that of overall grades. In Model 2, course setting was added as an independent variable, and the results showed that course setting had a significant positive effect on employability (β = 0.272, *P* < 0.01), i.e., the more reasonable course setting was, the stronger the employability of college students was. Hypothesis 1 was supported. In Model 3, course teaching was added as an independent variable, and the results showed that course teaching also had a significant positive effect on employability (β = 0.311, *P* < 0.01), i.e., the higher the level of college course teaching, the stronger the employability of college students. Hypothesis 2 was supported. In Model 4, club activities were added as an independent variable, and the results showed that club activities also had a significant positive impact on the employability of college students (β = 0.304, *P* < 0.01). When club activities were more plentiful, there was a more significant increase in the employability of college students. Hypothesis 3 was confirmed. Model 5 added employment guidance and the results showed that college employment guidance activities also had a significant positive impact on college students’ employability (β = 0.289, *P* < 0.01). College students’ employability was strengthened with the improvement of employment guidance activities, and Hypothesis 4 was supported.

To further analyze the relationship between the main variables and the effect on the dependent variable, we conducted a regression analysis with the four main variables simultaneously as independent variables. The result shows that the influence coefficient of course teaching and employment guidance is still the highest, and the explanatory power of R2 reaches 20.6%. This shows that the overall influence of the principal variable on the dependent variable is unchanged.

#### Common Method Bias

This article adopted two ways to avoid the common method bias: precontrol and postcontrol. In precontrol, the order of variables in the questionnaire was arranged randomly to ensure that respondents fill in the questionnaire anonymously. In postcontrol, all variables were analyzed by exploratory factor analysis mainly through the single-factor test, and the results showed that all factors accounted for 60% of the total variables. Therefore, there is no common method bias in this article.

## Discussion

Based on human capital theory and social capital theory, this study investigated the influence of college factors on college students’ employability, and the results show that among the university factors, course setting, course teaching, club activities, and employment guidance have a positive correlation with college students’ employability, among which course teaching and club activities have the most critical influence on college students’ employability. In addition, from the viewpoint of individual college students, the overall grades of college students, and the time of participating in the internship are also closely related to their employability, i.e., college students with good overall grades and long internship time should also have stronger employability.

### Theoretical Implications

The main results of this study have several important theoretical implications.

First, the employability constructs of college students were inconsistent, and few pieces of literature included information acquisition ability and career planning ability into the employability dimension, which were included in this study. The results indicated that the employability constructs had good reliability and validity. Therefore, this study further argued for the logical relationship between the employability concept and the employability construct.

Second, the antecedent variables of college students’ employability were inconsistent and using social capital theory, two antecedent variables were added to this study: club activities and employment guidance. The results showed that the coefficient of influence of club activities (β = 0.304, *P* < 0.01) ranked second after course instruction (β = 0.311, *P* < 0.01) on college students’ employability, which indicates that club activities have a very important influence on college student’ employability. College students’ participation in club activities cannot only enhance their practical ability but also enhance their communication and teamwork ability, thus improving their employability. The coefficient of the influence of employment guidance on college students’ employability (β = 0.289, *P* < 0.01) ranks third, which indicates that employment guidance is also important for college students’ employability improvement. College students’ participation in employment guidance activities can improve their ability of information acquisition and career planning, thus, enhancing their employability. Therefore, this study provides a new perspective for the study of employability and offers new research findings in this field.

Third, based on human capital theory, this study considered individual college students’ factors, and the results showed that there was a positive correlation between individual college students’ overall grades (β = 0.113, *P* < 0.01) and participation in internships (β = 0.281, *P* < 0.01) and college students’ employability. The more outstanding the overall performance of individual college students, the stronger their own employability. The longer the duration of participation in an internship, the stronger their own employability. The improvement of college students’ overall performance was more closely related to the input of course teaching and the reasonableness of the course setting. Therefore, the research results also indirectly confirm the key role of course setting and course teaching in improving the employability of college students.

### Practical Implications

Based on the college perspective, this study explores the factors influencing the enhancement of college students’ employability, and the findings have important practical implications for higher education.

First, the conclusion of the study points out that the rationality of the course setting helps to enhance the employability of college students. Therefore, the college should actively promote course reform and improve the course setting. For one thing, the course setting should be oriented to market demand, innovate talent training mode, and develop a forward-looking and practical course system. For another, scientific allocation of teaching time for basic college courses, professional courses, and practical courses is required. Among them, the professional course setting focuses on improving the professional skills of college students, and the social practice course setting focuses on improving the social practice ability of college students.

Second, this study concludes that the quality of course teaching helps to improve the employability of college students. Therefore, teaching quality control should be strengthened in the process of implementing the teaching plan. It is required to improve the course teaching process management and full management by the course teaching supervision office, from teaching faculty construction to course teaching implementation to teach quality assessment. Besides, it is necessary to improve the teachers’ assessment system and increase the weight coefficient, of course, teaching quality assessment, so that teachers will devote more time and energy to course teaching work. In addition, the college should encourage teachers to adopt a variety of teaching methods for course teaching, avoid indoctrination, and authoritative teaching mode, should focus on students’ enthusiasm and initiative, and actively promote heuristic teaching mode, such as through case teaching, to shorten the emotional distance between teachers and students as much as possible.

Third, this study concludes that college students’ active participation in club activities helps to improve their employability. Therefore, colleges should actively build platforms for club activities, college-enterprise cooperation, and social practice. For one thing, colleges should create a variety of club activities organizations to provide diversified choices for college students. At the same time, the college should actively carry out employment market research and build a college-enterprise cooperation platform according to the employment market demand, to provide more social practice opportunities for college students. For another, the college should improve the supporting facilities to ensure the benign operation of the practice platform. For example, it should improve the management system of clubs, provide funds for club activities, encourage students to participate in internships in the college-enterprise cooperation platform, and provide basic life support.

Fourth, this study concludes that college employment guidance activities help to enhance the employability of college students. Therefore, it is essential to improve the employment guidance system. For one thing, colleges should actively offer employability training courses, focusing on employment cognitive education, so that college students have a clearer understanding of the current employment situation and employment policies, plan their careers scientifically, and improve the comprehensive quality of college students’ employment. For another, the college should introduce social power to carry out employment guidance activities, including senior human resource experts from large foreign-funded enterprises, state-owned enterprises, and scientific and innovative growing enterprises, who can give lectures and provide one-to-one employment guidance services.

### Limitations and Future Directions

This study has some limitations. First, the sample data of this study are only limited to Anhui provincial universities, which slightly limits the generalizability of our findings. Future research can further verify our findings by expanding the sample source and sample size and analyzing the generalizability and applicability of the findings in different situations.

Second, the construct measurement scales in this study are mainly generalized based on previous studies by referring to some mainstream journal literature. Although these scales passed a series of reliability and validity tests, some scales measure design indicators that are only qualitative but not quantitative, such as the employability secondary interview (1 = not relevant, 2 = somewhat relevant, 3 = very relevant, and 4 = highly relevant), and suffer from a lack of discrimination. Future research needs to further develop and validate the scales, especially in terms of content validity.

Third, the sample of this study has variability, especially the quality of the college students’ group itself, and it is difficult to control effectively in the experiment. Therefore, the regression model derived from this study is not very effective, such as *R*^2^ and univariate analysis are relatively low in terms of statistical significance. In fact, there are many factors influencing college students’ employability, and this study only conducted a preliminary exploratory study on four factors: course setting, course teaching, club activities, and employment guidance.

Fourth, this study considers the initial employability of college students, and takes getting a job as the performance of employability but does not consider the quality of college students’ employment. Future research focuses on the relevance of university factors to college students’ employability and college students’ employment quality.

## Conclusion

Based on human capital theory and social capital theory, this study explored the influence of university factors on college students’ employability enhancement. The results show that among the university factors, course setting, course teaching, club activities, and employment guidance are positively correlated with college students’ employability, among which course teaching and club activities have the most critical influence on college students’ employability. In addition, from the perspective of individual college students, the overall grades of college students and the length of their internship are also closely related to their employability. In other words, if students have good overall grades and longer internship time, their employability will be stronger.

## Data Availability Statement

The original contributions presented in the study are included in the article/supplementary material, further inquiries can be directed to the corresponding author/s.

## Author Contributions

Y-CZ proposed the research hypothesis and research design and analyzed the experimental data. YZ contributed to questionnaires and collected data. X-LX combed the literature. J-BL analyzed the experimental results. R-BZ designed the framework of the manuscript and discussed the experimental results. All authors contributed to the article and approved the submitted version.

## Conflict of Interest

The authors declare that the research was conducted in the absence of any commercial or financial relationships that could be construed as a potential conflict of interest.

## Publisher’s Note

All claims expressed in this article are solely those of the authors and do not necessarily represent those of their affiliated organizations, or those of the publisher, the editors and the reviewers. Any product that may be evaluated in this article, or claim that may be made by its manufacturer, is not guaranteed or endorsed by the publisher.
